# Unveiling phytoconstituents and the anti-inflammatory potential of *Crassula tetragona* L. in ulcerative colitis: A focus on the PPARγ/SIRT1 axis

**DOI:** 10.1007/s10787-025-02049-6

**Published:** 2025-12-06

**Authors:** Mona A. Raslan, Rehab F. Abdel-Rahman, Hany M. Fayed, Marawan A. Elbaset, Rehab F. Taher

**Affiliations:** 1 Pharmacognosy Department, Pharmaceutical and Drug Industries Research Institute, National Research Centre, Dokki, Giza, 12622 Egypt; 2 Pharmacology Department, Medical Research and Clinical Studies Institute, National Research Centre, Giza, 12622 Egypt; 3 Natural Compounds Chemistry Department, Pharmaceutical and Drug Industries Research Institute, National Research Centre, Dokki, Giza, Egypt; 4 Faculty of Pharmacy, Pharmacognosy Department, Egyptian Chinese University, 14 Abou Ghazalh, Mansheya El-Tahrir, Ain Shams, Cairo Governorate, 4541312 Egypt

**Keywords:** Anti-inflammatory, *Crassula tetragona* L., Phytochemical, Phenolics, Ulcerative colitis

## Abstract

*Crassula* species are traditionally used and possess anti-inflammatory properties, but *Crassula tetragona* L. remains largely unexplored. This study intended to characterize *C. tetragona* aerial parts’ phytoconstituents and assess its anti-ulcerative potential via the PPARγ/SIRT1 pathway. Aerial parts of *C. tetragona* were extracted using n-hexane (CT1) and 70% aqueous methanol (CT2). Phytoconstituents were profiled by HPLC–ESI–MS/MS (negative ion mode), and phenolics were quantified by MRM-LC–ESI–MS/MS. Column chromatography and NMR were used to separate and identify the compounds. Ulcerative colitis (UC) was induced in rats by intrarectal acetic acid (AA). Animals were assigned into six groups: control group: orally received vehicle for 7 days, UC control group: orally received vehicle for 7 days, and a rectal infusion of 2 mL AA (4% v/v in saline) on the 8th day, 4 treated groups: received CT1 (200 and 400 mg/kg/day), or received CT2 (200 and 400 mg/kg/day), once daily for 7 days by oral gavage and 2 mL AA (4% v/v in saline) on the 8th day. HPLC–ESI–MS/MS identified 66 constituents, including 37 novel compounds, with CT2 exhibiting higher phenolic content. Naringenin, gallic acid, and quercetin were predominant. Five phenolic compounds were isolated from the bioactive extract CT2. Both CT1 and CT2 reduced AA-induced tissue damage, lowered inflammatory markers (calprotectin, CRP, TNF-α, IL-6), improved oxidative stress (reduced MDA, increased GSH, SOD), and upregulated SIRT1 and PPARγ. These results suggest *C. tetragona* attenuates UC via the SIRT1/PPARγ pathway, indicating its therapeutic potential.

## Introduction

Inflammatory bowel disease (IBD) is a chronic, recurrent, idiopathic gastrointestinal inflammatory illness (Ghouri et al. 2020) thought to be brought on by a modified immunological reaction to the gut microbiota. Additionally, its etiopathogenesis is shaped by hereditary and external elements (Rafeeq et al. 2021). The two principal types of IBD are Crohn’s disease (CD) and ulcerative colitis (UC) (Kővári et al. 2022). CD can affect the whole gastrointestinal system, whereas UC just affects the colon and rectum (Baima et al. 2023)**.** Ulcerative colitis is mainly characterized by stomach pain, diarrhoea, passing stools more often, rectal bleeding (including blood in the stool and colonic haemorrhage), and weight loss (Mowat et al. 2011)**.** Globally, an estimated 5 million cases of UC were reported in 2023, and the incidence is continually increasing (Le Berre et al. 2023)**.** This emphasizes the value of research in healthcare advances and prevention (Wang et al. 2024)**.** Unfortunately, the main goal of the medical care that is now provided for chronic UC is to prevent complications from the condition (Abdelhamid et al. 2022). Furthermore, the clinical results of current treatments are frequently insufficient (Zohny et al. 2022)**.** Furthermore, patients who fail to respond to existing therapies frequently require hospitalization. Thus, developing new drugs with distinct mechanisms of action is crucial for improving treatment outcomes (Abdelhady et al. 2023).

As UC develops, a number of intricate biological processes interact to contribute to the pathophysiology of the illness (Nasr et al. 2022). UC exacerbates the disease by dysregulating a number of important cellular processes. Silent information regulator 1 (SIRT1) belongs to the family of mammalian sirtuin proteins. SIRT1 plays a crucial part in preventing chemically induced colitis (Zhang et al. 2019). Numerous clinical and experimental studies have demonstrated that SIRT1 levels are markedly reduced in UC. Crucially, activating SIRT1 has been found to significantly ease the signs of colitis (Sharma et al. 2014; Caruso et al. 2014), and SIRT1 activators significantly reduced the symptoms of colitis (Devi et al. 2020). Peroxisome proliferator-activated receptors (PPARs) are another important component that influences gut homeostasis by influencing both innate and adaptive immune responses in the lamina propria (Riccardi et al. 2009). Because the PPAR isoforms are mainly found in the human colonic epithelium, activating them directly leads to the suppression of nuclear factor kappa B (NF-κB) signaling. This action, in turn, reduces the output of inflammatory cytokines (Suárez et al. 2012). In order to treat UC, several studies have shown that medications work by interacting with PPARγ is a useful target for UC treatment (Arafa et al. 2020).

Recent research heavily features natural products, primarily because they possess anti-inflammatory capabilities and are generally non-toxic. Evidence confirms that certain active herbal components can successfully alter the inflammation linked to human IBD and experimental models of colitis (Guo et al. 2017). Previous research on various plants in the Crassulaceae family has documented the isolation of a wide array of compounds, including phenolic components, sterols, hydrocarbons, ascorbic acid, organic acids, triterpenoids, and bufadienolides (Eid et al. 2018). Various species within this plant family are reported to possess a broad spectrum of therapeutic and biological activities. These properties include effects that are anti-inflammatory, anti-oxidant, anti-cancer, anti-ulcer, and antimicrobial. Additionally, they show potential as pain relievers analgesic/anti-nociceptive, as well as having liver-protecting, antidiabetic, and even antimalarial impacts, among others (El-Hawary et al. 2016a; Hassan et al. 2021). For the treatment of gastric ulcers and haemorrhages, *Orostachys japonicus* (Crassulaceae) plants have been utilized (Nugroho et al. 2012). *Crassula tetragona* L. (Family: Crassulaceae), a succulent plant native to South Africa and Mozambique, has not been extensively studied phytochemically or pharmacologically. The genus *Crassula* is particularly abundant in phytocompounds such as phenolics, triterpenes, and sterols (Hassan et al. 2021). Several pharmacological activities were documented for *Crassula* species like anticonvulsant, anti-oxidant, anti-inflammatory, antimicrobial, and anti-arthritic activities (Amabeoku et al. 2014; El-Hawary et al. 2016b, a; Hassan et al. 2021). *Crassula* species were traditionally used for diarrhoea, epilepsy and diabetes (Hassan et al. 2021). This study aims to characterize the phytoconstituents of *C. tetragona* L. unflowering aerial parts and assess its potential therapeutic benefits against UC in an animal model, focusing on the underlying mechanisms involving the PPARγ/SIRT1 pathway.

## Experimental section

### Bioactivity study

#### Chemicals

Acetic acid (AA) was purchased from CID Pharmaceutical Co. in Giza, Egypt. All other chemical employed in the study met the strict criteria of being analytical grade and possessing the utmost purity.

#### Experimental animals

Male Wistar rats weighing 150 to 170 g were obtained from the National Research Centre’s Animal Facility in Egypt. The rats were kept in standard conditions with free access to both food and water. All procedures were conducted with the formal approval of the National Research Centre’s Ethical Committee for Medical Research (Approval No.: MREC-13060133).

#### Induction of colitis

To prepare for colitis induction, the Wistar rats were fasted for one day but still had unlimited access to water. The rats were then anaesthetized using 100/mg/kg of Ketamine administered intraperitoneally (*i.p*.). Colitis was then induced by intrarectally administering 2 mL of 4% v/v acetic acid (AA) solution (in saline) via a polyurethane tube inserted 4.5 cm into the colon. To prevent the solution from leaking out, the rats were held in the Trendelenburg position during the instillation and for 1 min afterward (Bezerra et al. 2017).

#### Experimental design

Forty-eight rats were divided randomly into 6 groups of 8 rats each. Group I (Control rats): received only the vehicle (distilled water, DW) orally for eight days and a rectal infusion of saline on 8th day. Group II (AA group): received 2 mL AA (4% v/v in saline) rectally at the 8th day. Group III and IV (CT1-200 and CT1-400 group + Control): received CT1 (200 and 400 mg/kg/day) once daily for 7 days by oral gavage and 2 mL AA (4% v/v in saline) on the 8th day. Group V and VI (CT2-200 and CT2-400 group + Control): received CT2 (200 and 400 mg/kg/day) once daily for 7 days by oral gavage and 2 mL AA (4% v/v in saline) on the 8th day.

#### Sample preparation

Forty-eight hours after inducing colitis with acetic acid, the rats were anaesthetized using thiopental sodium (40 mg/kg, ip). The colonic segments were then immediately removed, trimmed of fat, and rinsed in cold saline. These cleaned segments were then processed for the following three distinct analyses: macroscopic scoring, biochemical testing and histological examination. Macroscopic scoring was performed on colonic sections. Other sections were used to prepare colon homogenates, which were frozen at -80 °C for later biochemical testing. A third portion was preserved in 10% neutral buffered formalin for subsequent histological examination (microscopic analysis).

#### Colonic wet weight assay

The extent of edema (swelling) and the overall severity of colitis were determined by first weighing the distal 8 cm section of the rat colon (Zhou et al. 2001). Following this, wet weight-to-length ratio (g/cm) of the colon specimens were calculated to get a standardized measure of inflammation (Abdel-Rahman et al. 2015).

#### Macroscopic colonic damage scoring

To visually assess the extent of mucosal damage, the colon tissues were cut open lengthwise. The severity of the lesions observed on the inner surface of the colon (macroscopic) was then graded using a 0 to 4 scale (Table 1) (Millar et al. 1996).

**Table 1:** Macroscopic Scoring of Colon Sections

Score	Criterion
0	No visible changes or signs of macroscopic damage
1	Only redness (mucosal erythema) is present
2	Mild swelling (edema), minor bleeding, or small erosions are observed
3	Features include moderate swelling (edema) alongside bleeding ulcers or erosions
4	The most severe damage, characterized by tissue death (necrosis), significant swelling (edema), and severe, widespread ulceration or erosion

#### Measurement of serum CRP and CALP

The concentrations of C-reactive protein (CRP) and CALP (Calprotectin) in the serum were measured using commercially available ELISA kits. The CRP kit was sourced from BT-LAB (Cat. No. E0053Ra, BT-LAB, Shanghai, China), and the CALP kit was from Elabscience® (Cat. No. E-EL-R2389, Elabscience®, Wuhan, China), with all assays strictly adhering to the manufacturers’ instructions.

#### Measurement of colon TNF-α, IL-6 and IL-10

The study quantified the colon concentrations of tumor necrosis factor-alpha (TNF-α), interleukin-6 (IL-6), and interleukin-10 (IL-10) were assessed biochemically using rat-specific ELISA kits (BioLegend, Cat. No. 438204, San Diego, USA), (CLOUD-CLONE CORP., Cat. No. SEA079Ra, Katy, TX 77494, USA), and (BT-LAB, Cat. No. E0108Ra, Shanghai, China), respectively.

#### Assessment of colon oxidative stress markers

The study measured the activity of reduced glutathione (GSH), malondialdehyde (MDA) level, and superoxide dismutase (SOD) activity in the colon tissue using an ELISA kit from BioVision, Milpitas, USA (Cat # K464-100, Cat # K739-100, and Cat #K335-100), respectively.

#### Histopathology examination

The harvested colon tissues of two rats per group were fixed in 10% neutral buffered formalin for 24 h, washed with tap water, dehydrated, cleared in xylene, routinely prepared to obtain 5–6 micron-thick paraffin-embedded sections and stained with H&E stain for light microscopy.

For histological study, colon tissues from two rats per group were first fixed in 10% neutral buffered formalin for 24 h. They were then thoroughly washed, dehydrated, and cleared in xylene. Next, the samples were embedded in paraffin and cut into very thin (5 to 6 micron thick) sections. Finally, these sections were stained with H&E (Hematoxylin and Eosin) for visual analysis.

#### Quantitative real-time PCR analysis

The gene expression levels of SIRT1 and PPAR-γ (with GAPDH mRNA serving as the reference gene) expression were quantified using quantitative Reverse Transcriptase-Polymerase Chain Reaction. First, RNA was extracted from the colon tissue using a kit from Macherey–Nagel GmbH & Co. (KG- Germany.) and then checked for purity (A260/A280 ratio) and concentration via spectrophotometry (dual wave length Beckman, Spectrophotometer, USA). The prepared RNA samples were stored at –80 °C. The qRT-PCR itself was performed using primers supplied from Bioline, a median life science company, UK (SensiFAST™ SYBR® Hi-ROX One-Step Kit, catalog no.PI-50217 V), and the resulting gene expression data was calculated and expressed using the 2 − ΔΔCT method. Primer sequence for the studied target genes (PPARγ&Sirt-1) and reference housekeeping gene (GAPDH) were shown in Table 2.

**Table 2:** Primer’s Sequence of All Studied Genes

Gene symbol	Primer sequence from 5′- 3′
PPARγ	F: CGAGTGCCGAGTCTGTGGGGATAA R: ATGGTGATTTGTCTGTTGTCTTTC XM_039107130.2
Sirt-1	F: GGGATCTCTAGGCCCAGTTC R: CTTTGGGGAGAGGGGGAC NM_001107073.1
GAPDH	F: CACCCTGTTGCTGTAGCCATATTC R: GACATCAAGAAGGTGGTGAAGCAG NM_001394060.2

#### Statistical analysis

All experimental outcomes are presented as means ± standard error (SE). The tissue analyses specifically involved six samples per group. For comparing multiple groups, one-way analysis of variance one-way analysis of variance (ANOVA) were utilized, followed by the Tukey test at *p* ≤ 0.05. The lesion scores were analysed separately using the non-parametric Kruskal–Wallis test, followed by the Mann–Whitney test. All data processing and graphical generation were performed using GraphPad prism® software (version 6.00 for Windows, San Diego, CA, USA).

### Phytochemical study

#### General

Nuclear Magnetic Resonance (NMR) spectra (500 MHz for ^1^H and 125 MHz for ^13^C) were collected using a JEOL Spectrometer and processed with Delta NMR Software. For compound isolation, column chromatography utilized Diaion® HP-20 (Sigma-Aldrich Chemie GmbH, Germany), Silica gel G (E. Merck, Darmstadt, Germany) 60 mesh, and Sephadex LH-20 (E Merck) as stationary phases. Thin-layer chromatography (TLC) was performed with Silica gel aluminium sheets G 60 (F254-Merck) and spots were located using a UV lamp. All solvents used for extraction and chromatography were analytical grade (from El-Gomhouria, Egypt, and Sigma-Aldrich, St. Louis, MO, USA), while HPLC-grade acetonitrile and formic acid were also obtained from Sigma-Aldrich. Highly pure water was prepared using a Milli-Q system (Millipore, USA).

#### Plant material

*Crassula tetragona* L. (Family: Crassulaceae) unflowering aerial parts were collected in spring 2023 from Helal Cactus farm, Al Mansoureyah, Imbaba, Giza Governorate, Egypt. The plant was officially identified and authenticated by scientist Treiz Labib at the Orman Garden herbarium in Cairo, Egypt. A voucher specimen (M 192) has been permanently stored in the National Research Centre Herbarium. For experimental use, the collected plant material was then air-dried, ground into powder, and stored in a dry container.

#### Extraction

Five hundred grams of the unflowering aerial parts of *C. tetragona* L. unflowering aerial parts were subjected to a two-step extraction process. First, the plant material was soaked four times overnight in n-hexane until fully exhausted. The resulting n-hexane extract (CT1) was then evaporated under vacuum, yielding 9 g of crude extract. Next, the remaining plant material was air-dried for three days. This dried material was then soaked four times in 70% aqueous methanol until exhausted. The resulting combined methanolic extract (CT2) was also evaporated under vacuum, yielding a much larger quantity of crude extract, 65 g.

#### Untargeted high-performance liquid chromatography coupled with electrospray ionization mass spectrometry (HPLC–ESI–MS/MS) analysis

A one-milligram sample was initially dissolved in 1 mL of 100% MeOH. To prepare it for analysis, the solution was sonicated for two minutes, then centrifuged at 13,000 rpm for three minutes) to effectively remove all insoluble particles. The clean supernatant was then collected for the next steps. The separation and detection were performed using a sophisticated LC–MS/MS system (ExionLC AC coupled with a SCIEX Triple Quad 5500 +), employing chromatographic separation followed by mass spectrometry detection in the negative electrospray ionization ESI mode. The separation utilized an Ascentis® Express 90 Å C18 (2.1 × 150 mm, 2.7 µm), with a mobile phase consisting of A: 5 mM ammonium formate (pH 8); B: acetonitrile (LC grade). A gradient program was run over 30 min at a flow rate of 0.3 mL/min, with a 5 µL injection volume. Gradient: 5% B (0–1 min), 5–100% B (1–20 min), 100% B (20–25 min), 5% B (25.01–30 min). Key Mass Spectrometry parameters included a source temperature of 500 °C, an ion spray voltage of -4500 V, and a declustering potential of -80 V. Data was acquired using the EMS-IDA-EPI scan mode and subsequently processed using PeakView ® software (AB Sciex).

#### Quantitative determination of phenolics using LC–ESI–MS/MS multiple reactions monitoring mode (MRM)

Standard (1 mg/mL) solutions of the phenolic standards were prepared and diluted to 10 μg/mL for storage, with a 200-ppb mixture used for calibration. Plant extract (3–5 mg) was dissolved in 80% methanol, sonicated, and filtered before analysis. The samples were then analysed by LC–MS/MS using an ExionLC coupled to a SCIEX Triple Quadruple 5500 + . Chromatographic separation employed a ZORBAX Eclipse Plus C18 (4.6 × 100 mm, 1.8 μm) column, a formic acid in water (A)/acetonitrile (B) mobile phase gradient (0.8 mL/min), and 3 μL injection. Gradient elution:0–1 min: 2% B, 1–21 min: 2–60% B, 21–25 min: 60% B, 25.01–28 min: 100% B. Detection utilized Electrospray Ionization (ESI) in both positive and negative modes.

#### Isolation

Fifty-five grams of the CT2 extract were separated using a Diaion HP-20 column (1 kg) column chromatography (CC). The fractions were eluted using a gradient solvent system that progressed from pure water H_2_O to pure methanol MeOH. The collected fractions were monitored for content using both silica gel and cellulose plates via TLC. For the silica TLC plates, the following solvent systems CH_2_Cl_2_-MeOH (8:2) and EtOAc–MeOH (98:2) were used. BAW ( n -butanol: Acetic acid: H2O; 4:1:5; upper phase) and 15% Acetic acid were used to develop cellulose TLC plates. The silica plates were visualized by a two-step process: UV light inspection, followed by spraying with vanillin-H_2_SO_4_ reagent and subsequent heating to 100 °C. The cellulose plates were examined under UV lamb first and then exposed to ammonia vapours and re-visualized under UV lamb. Identical fractions were pooled to give 4 main fractions. Fractions A (15 g) and C (9 g) resulted from the main column with H_2_O-MeOH (75:25) and (25:75), respectively, were selected for further isolation. Using silica gel CC (300 g) and solvent system CH_2_Cl_2_-MeOH (1:0–0:1)., fraction A yielded 40 subfractions (50 mL). According to the visualization of cellulose TLC plates, the subfractions were grouped into 6 similar subfractions. Subfraction 2A, eluted with a 9:1 mixture of CH_2_Cl_2_ and MeOH, was separated by preparative paper chromatography on Whatman No. 3 MM cellulose sheets using 15% acetic acid in water as the mobile phase. The preparative paper chromatography of subfraction 2A yielded 5 bands. Band 2 was cut into pieces and extracted using 70% aqueous methanol three times. Band 2 yielded compound **42** (12 mg) using Sephadex LH-20 CC and EtOH-H_2_O (5:5) as eluent. Band 5 was cut into pieces and extracted using 70% aqueous methanol three times. Band 5 yielded compounds **41** (12 mg) and **44** (15 mg) using Sephadex LH-20 CC and EtOH-H_2_O (5:5) as eluent. Fraction C was applied on silica gel CC (300 g) and eluted with solvent system CH_2_Cl_2_-MeOH (1:0–0:1), yielding 30 subfractions (50 mL). After visualization of cellulose TLC plates, the subfractions were grouped into 12 identical subfractions. Subfraction 4C eluted with an 8:2 mixture of CH_2_Cl_2_ and MeOH, were applied to Sephadex LH-20 CC using MeOH-H_2_O (1:0–0:1) for elution. The fractions eluted at 20% MeOH in H_2_O were further refined using Sephadex LH-20 and EtOH-H_2_O (2:8) to yeild compound **46** (13 mg). While the fractions eluted at 15% MeOH in H_2_O were further refined using Sephadex LH-20 and EtOH-H_2_O (2:8) to afford compound **38** (15 mg).

*Quercetin 3,7-dimethyl ether (3',4',5-trihydroxy-3,7-dimethoxyflavone)* **42.**

Yellow needles; C_17_H_14_O_7_; ESI–MS m/z [M-H]^−^; ^1^H-NMR (500 MHz, acetone-d6) δ: 12.73 (1H, *s*, 5-OH), 8.03 (1H, *d*, *J* = 2.1, H-2`), 7.13 (1H, *dd*, *J* = 7.7, 2.1, H-6`), 7.00 (1H, *d*, *J* = 7.7, H-5`), 6.84 (1H, *d*, *J* = 2.2, H-8), 6.24 (1H, *d*, *J* = 2.2, H-6), 3.79 (3H, *s*, 7-OMe), 3.69 (3H, *s*, 3-OMe). ^13^C-NMR (125 MHz, acetone-d6) δ: 183.4 (C-4), 165.8 (C-7), 161.1 (C-5), 156.9 (C-9), 156.2 (C-2), 148.3 (C-4`), 144.8 (C-3`), 137.6 (C-3), 122.1 (C-1`), 121.3 (C-6`), 115.8 (C-2`), 114.6 (C-5`), 97.9 (C-6), 91.7 (C-8), 59.3 (3-OMe), 55.5 (7-OMe) (Nantapap et al. 2017).

*(2S)-Eriodictyol (3',4',5,7-tetrahydroxyflavanone)* **41.**

Colourless needles; C_15_H_12_O_6_; ESI–MS m/z 287 [M-H]^−^; ^1^H-NMR (500 MHz, acetone-d6) δ: 2.71 (1H, *dd*, *J* = 12.1, 3.1 Hz, H-3*cis*), 3.14 (1H, *dd*, *J* = 16.9, 12.4 Hz, H-3*trans*), 5.38 (1H, *dd*, *J* = 12.7, 3.1 Hz, H-2), 5.92 (1H, *d*, *J* = 2.0 Hz, H-6), 5.93 (1H, *d*, *J* = 2.0 Hz, H-8), 6.84 (2H, *d*, *J* = 1.5 Hz, H-5`, H-6`), 7.00 (1H, *d*, *J* = 1.5 Hz, H-2`), 12.14 (1H, *s*, HO-5); ^13^C-NMR (125 MHz, acetone-d6) δ: 42.8 (C-3), 79.2 (C-2), 95.1 (C-8), 96.0 (C-6), 102.3 (C-10), 113.9 (C-2`), 115.2 (C-5`), 118.3 (C-6`), 130.6 (C-1`), 145.3 (C-3`), 145.7 (C-4`), 164.2 (C-5), 164.5 (C-9), 166.7 (C-7), 196.4 (C-4) (Buranasudja et al. 2022).

*Padmatin (Taxifolin 7-methyl ether)* **44.**

Colourless needles; C_15_H_12_O_6_; ESI–MS m/z 317 [M-H]^−^; ^1^H-NMR (500 MHz, acetone-d6) δ: 3.86 (1H, *s*, 7-OMe), 5.00 (1H, *d*, *J* = 12.7, H-3), 5.38 (1H, *d*, *J* = 12.7, H-2), 5.92 (1H, *d*, *J* = 2.0 Hz, H-6), 5.93 (1H, *d*, *J* = 2.0 Hz, H-8), 6.84 (2H, *d*, *J* = 1.5 Hz, H-5′, H-6′), 7.00 (1H, *d*, *J* = 1.5 Hz, H-2′), 12.14 (1H, *s*, HO-5); ^13^C-NMR (125 MHz, acetone-d6) δ: 55.6 (7-OMe), 74.9 (C-3), 82.4 (C-2), 93.2 (C-8), 94.8 (C-6), 100.0 (C-10), 112.9 (C-2`), 117.7 (C-5), 127.6 (C-1`), 151.3 (C-4`), 155.4 (C-3`), 164.9 (C-9), 165.2 (C-5), 168.4 (C-7), 194.8 (C-4) (Minh et al. 2022).

*3,5,7,4`,3``,5``,7``-Heptahydroxy-3`-O-4```-biflavanone* **46.**

Yellowish amorphous solid; C_30_H_22_O_12_; ESI–MS m/z 573 [M-H]^−^; 1H-NMR (500 MHz, acetone-d6) δ: 4.62 (1H, *d*, *J* = 11.5 Hz, H-3``), 4.69 (1H, *d*, *J* = 11.5 Hz, H-3), 5.05 (1H, *d*, *J* = 11.5 Hz, H-2``), 5.14 (1H, *d*, *J* = 11.5 Hz, H-2), 6.01 (4H, *d*, *J* = 2.4 Hz, H-6, H-8, H6``, H-8``), 6.87 (2H, *d*, *J* = 8.4 Hz, H-3```, H-5```), 7.50 (1H, *d*, *J* = 6.5 Hz, H-5`), 7.66 (1H, *dd*, *J* = 8.5, 2.1 Hz, H-6`), 7.79 (1H, *d*, *J* = 2.2 Hz, H-2`), 8.12 (2H, *d*, *J* = 8.6 Hz, H2```, H-6```), 11.95 (2H, *br s*, OH-3,OH-3``), 12.14 (2H, *s*, OH-7), 13.05 (1H, *s*, OH-5); ^13^C-NMR (125 MHz, acetone-d6) δ: 72.3 (C-3, C-3`), 83.7 (C-2, C-2``), 95.2 (C-8, C-8``), 98.8 (C-6, C-6``), 100.6 (C-10, C-10`), 114.9 (C-3```, C-5```), 115.1 (C-5`), 120.6 (C-2`), 124.3 (C-6), 128.3 (C-2```, C-6```), 129.5 (C1`), 130.0 (C-2```, C-6```), 150.6 (C-4```), 159.4 (C-4`), 164.2 (C-9, C-9``), 165.0 (C-5, C-5``), 166.7 (C-7, C-7``), 197.6 (C-4, C-4``) (Sievers et al. 1992; Pegnyemb et al. 2005).

*6```-hydroxylophirone B ([(2S,3R)-naringenin-(3β,3)-4,2`,4`-trihydroxychalcone]* **38.**

Yellow needles; C_30_H_22_O_9_; ESI–MS m/z 525 [M-H]^−^; 1H-NMR (500 MHz, acetone-d6) δ: 4.70 (1H, *d*, *J* = 12.0 Hz, H-α), 5.94 (1H, *d*, *J* = 12.0 Hz, H-β), 6.28 (1H, *s*, H-3`), 6.49 (1H, *d*, *J* = 8.7 Hz, H-5`), 6.87 (2H, *d*, *J* = 8.7 Hz, H-3``, H-5``), 6.96 (1H, *d*, *J* = 8.0 Hz, H-5), 7.27 (2H, *d*, *J* = 8.6 Hz, H-2``,H-6``), 7.52 (1H, *br s*, H-2), 8.07 (1H, *d*, J = 8.9 Hz, H-6`), 12.26 (1H, *s*, HO-6``), 13.03 (1H, *s*, OH-2`); ^13^C-NMR (125 MHz, acetone-d6) δ: 54.1(C-α`), 82.3 (C-β`), 95.0 (C-3```), 96.2 (C-5```), 102.1 (C1```), 105.1 (C-3`), 108.4 (C-5`), 114.8 (C-1`), 115.1 (C-3``, C-5``), 115.8 (C-5), 118.2 (C-α), 122.4 (C-3), 128.8 (C-1), 129.1 (C-1``), 129.7 (C-2``, C-6``, C-6), 131.2 (C-6`), 132.7 (C-2), 146.4 (C-β), 157.8 (C-4``), 159.4 (C-4), 164.4 (C-4`), 164.8 (C-6```), 166.4 (C-4``), 167.2 (C-2`), 191.9 (CO–c), 197.1 (CO–c) (Kaewamatawong et al. 2002).

## Results

### Bioactivity study

#### Effect on macroscopic lesion score and colonic wet weight assay

**Fig. 1:**
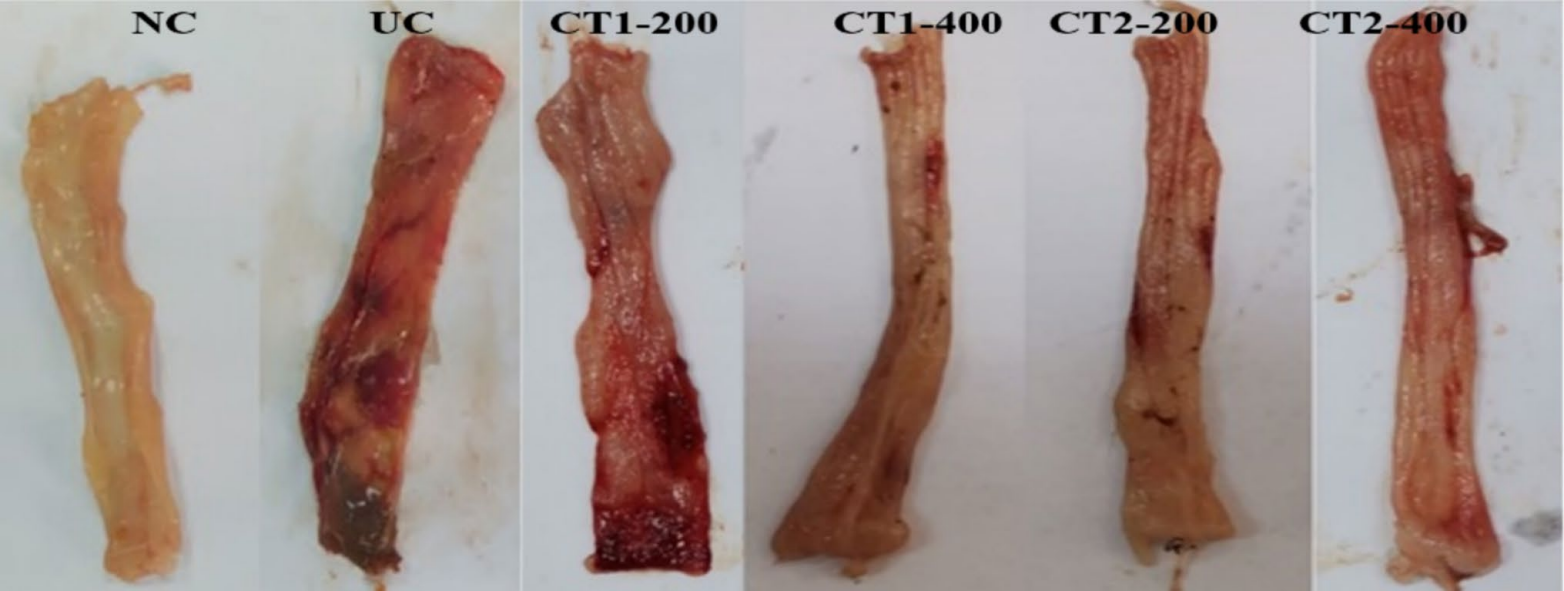
Macroscopic appearance of colon sections

Figure 1 provides a visual representation of the macroscopic view of the colon segments obtained from the rats. Figure 1 depicts the macroscopic appearance of rat colon segments. In the UC group, extensive colonic macroscopic ulcers and haemorrhagic lesions developed after intrarectal acetic acid instillation. However, CT1 and CT2 administered groups alleviated these macroscopic alterations in a dose-dependent way, with CT2 treated rats showing more favourable results at the two examined dose levels.

**Fig. 2:**
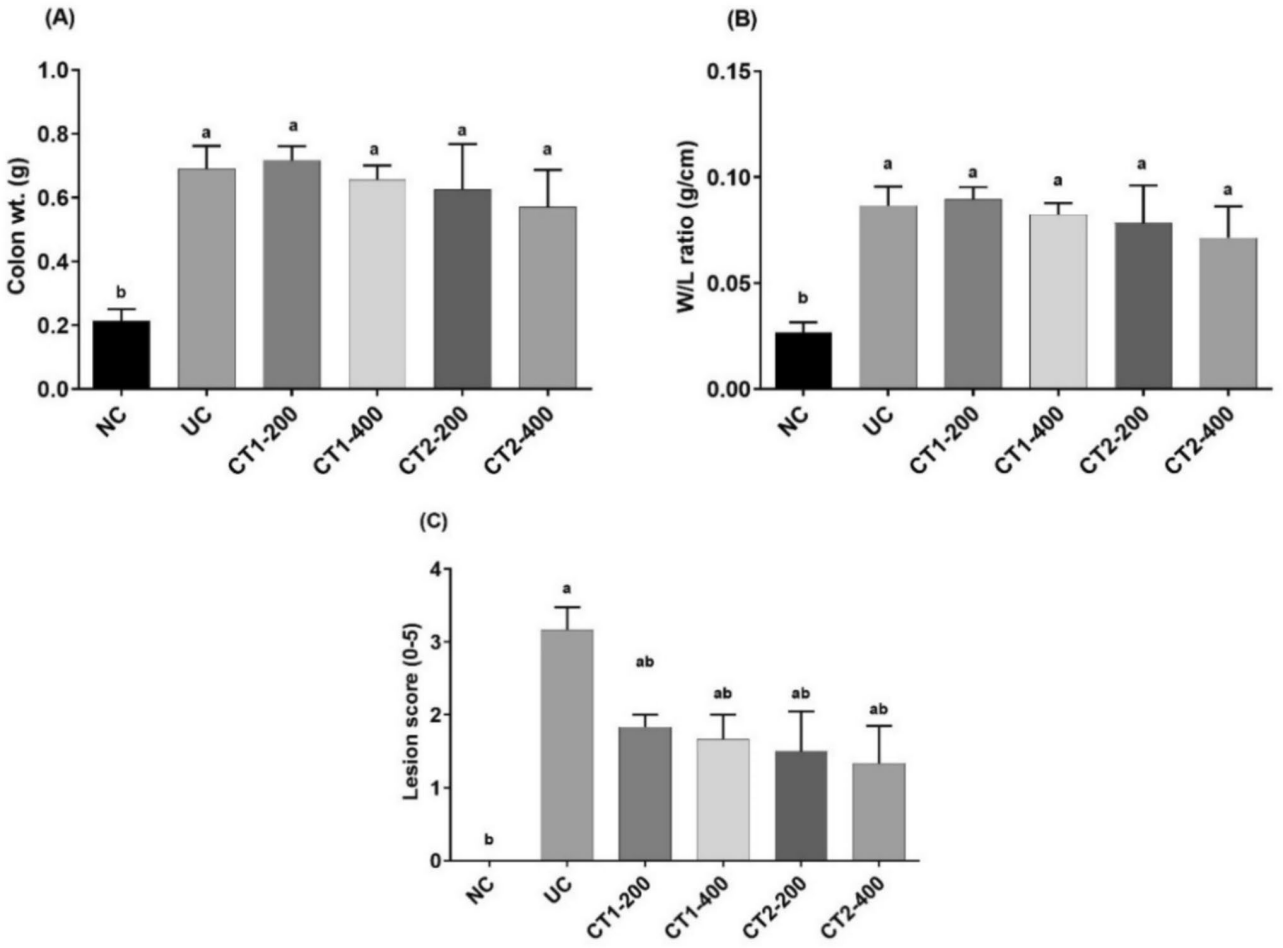
Effect on colon weight, W/L ratio and lesion score. Data presented as mean ± SEM (n = 6).^a^Statistically significant from the NC group at *P* ≤ 0.05.^b^Statistically significant from the UC group at *P* ≤ 0.05

Intrarectal acetic acid was used to trigger colitis, causing the colons of the ulcerative control group to become significantly heavier and larger relative to their length (higher W/L ratio) compared to the normal controls. Furthermore, the colon weight and wet weight/length ratio were significantly elevated by 228.571% and 218.519% than the normal control group, respectively. CT1 and CT2 treatment reduced increased colon weight and wet weight/length ratio in comparison to the ulcerative control group. Furthermore, the colon lesion score was significantly increased in the UC group compared to the NC animals. CT1 and CT2 treatments significantly reduced lesion score in comparison to UC rats (Fig. 2).

#### Effect on serum CRP and CALP

Data in Table 3 demonstrate that the UC group shows significantly higher levels of CRP (3.95 ± 0.11 ng/mL) and CALP (21.77 ± 0.80 ng/mL) compared to NC (CRP: 0.4 ± 0.02, CALP: 6.95 ± 0.30). Treatments CT1 and CT2, reduced both markers with CT2-400 showing the strongest efficacy in comparison to the UC group (*p* ≤ 0.05).

**Table 3:** Effect on serum CRP and CALP

Group	CRP (ng/mL)	CALP (ng/mL)
NC	0.4 ^b^ ± 0.02	6.95 ^b^ ± 0.30
UC	3.95 ^a^ ± 0.11	21.77 ^a^ ± 0.80
CT1-200	2.60 ^ab^ ± 0.12	15.98 ^ab^ ± 0.30
CT1-400	1.20 ^ab^ ± 0.07	12.05 ^ab^ ± 0.54
CT2-200	2.15 ^ab^ ± 0.15	11.46 ^ab^ ± 0.24
CT2-400	0.83 ^ab^ ± 0.06	8.98 ^ab^ ± 0.30

Data presented as mean ± SEM (n = 6)

^a^ Statistically significant from the NC group at *P* ≤ 0.05

^b^ Statistically significant from the UC group at *P* ≤ 0.05

#### Effect on colon TNF-α, IL-6 and IL-10

**Fig. 3:**
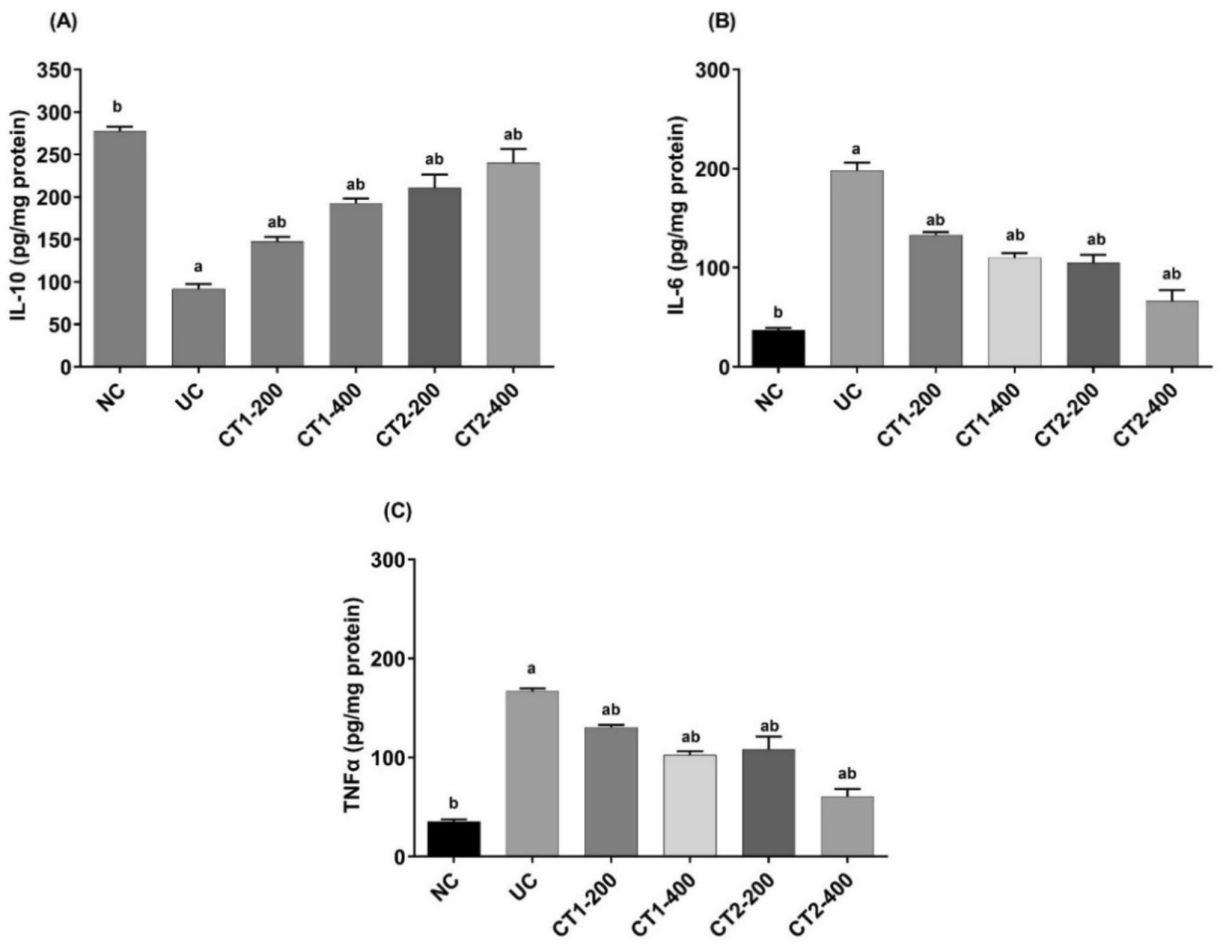
Effect on colon IL-10, IL-6 and TNF-α. Data presented as mean ± SEM (n = 6). ^a^Statistically significant from the NC group at *P* ≤ 0.05.^b^Statistically significant from the UC group at *P* ≤ 0.05

UC induction resulted in a significant decrease in IL-10 level (91.64 ± 23.64 vs. NC: 277.42 ± 51.34) (Fig. 3). Moreover, significant increase in IL-6 and TNFα (198.26 ± 8.67 and 166.90 ± 2.92, respectively) vs. NC (37.27 ± 2.08 and 35.57 ± 2.30, respectively). Treatment with CT1 and CT2, across both dose levels, resulted in a significant shift toward an anti-inflammatory state compared to UC group. This was demonstrated by a significant rise in IL-10 level, and a significant decrease in both IL-6 and TNFα (*p* ≤ 0.05).

#### Effect on colon oxidative stress markers

**Fig. 4:**
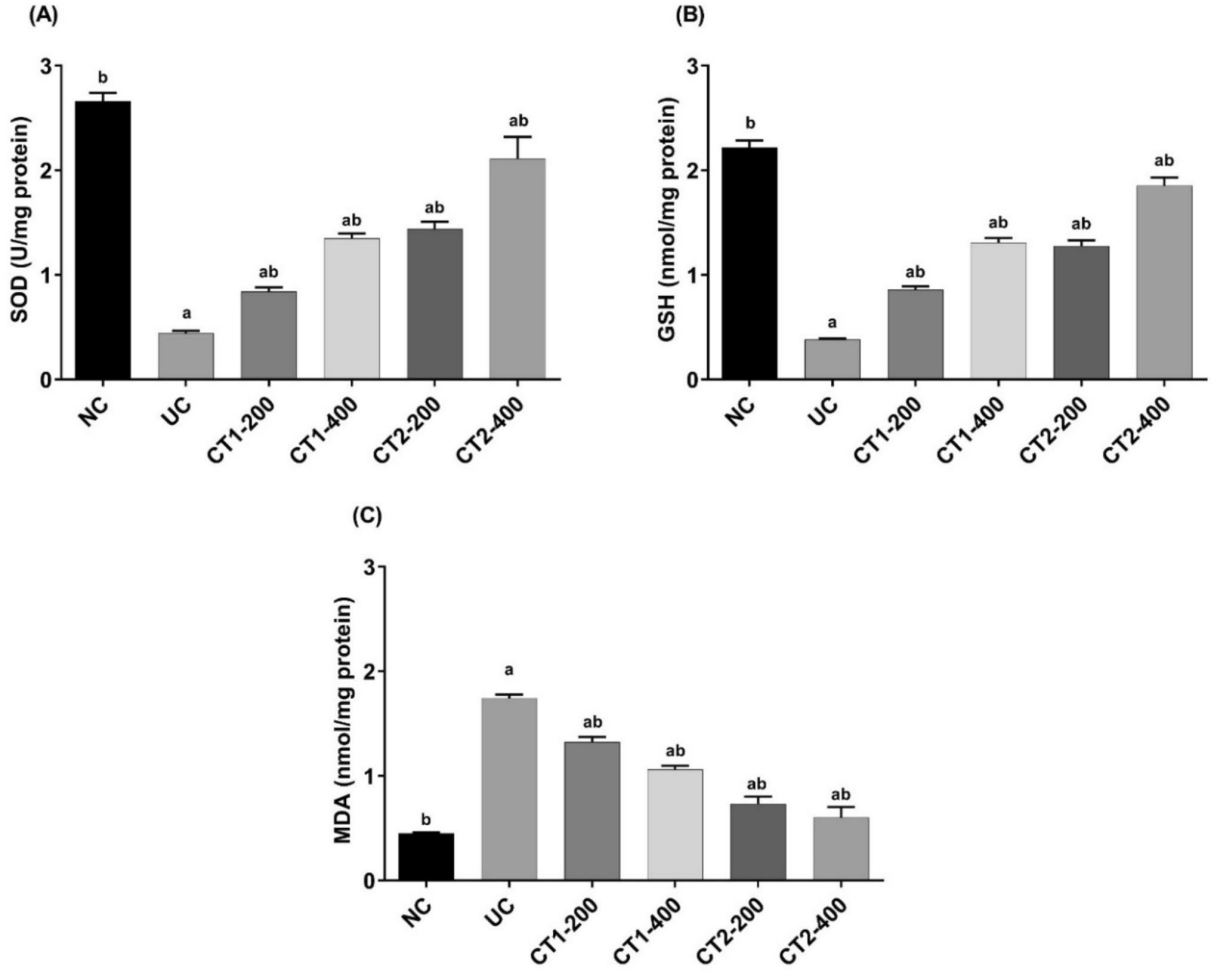
Effect on colon SOD, GSH and MDA. Data presented as mean ± SEM (n = 6). ^a^Statistically significant from the NC group at *P* ≤ 0.05.^b^Statistically significant from the UC group at *P* ≤ 0.05

Inducing ulcerative colitis increased oxidative stress in colon tissue, as shown in Fig. 4. In UC group, SOD (0.44 ± 0.02 U/mg protein) and GSH (0.38 ± 0.35 nmol/mg protein) levels were significantly lower compared to NC (SOD: 2.66 ± 0.12, GSH: 2.22 ± 0.40), while MDA (1.74 ± 0.03 nmol/mg protein) levels were significantly higher (vs. NC: 0.45 ± 0.01).

Treatment with CT1 and CT2, across both dose levels, resulted in significant improvement of the oxidative status. CT1-200, CT1-400, CT2-200 and CT2-400 significantly increased SOD and GSH activities, compared to UC rats, and significantly declined MDA levels in comparison to UC group.

#### Effect on colon histopathology examination

**Fig. 5:**
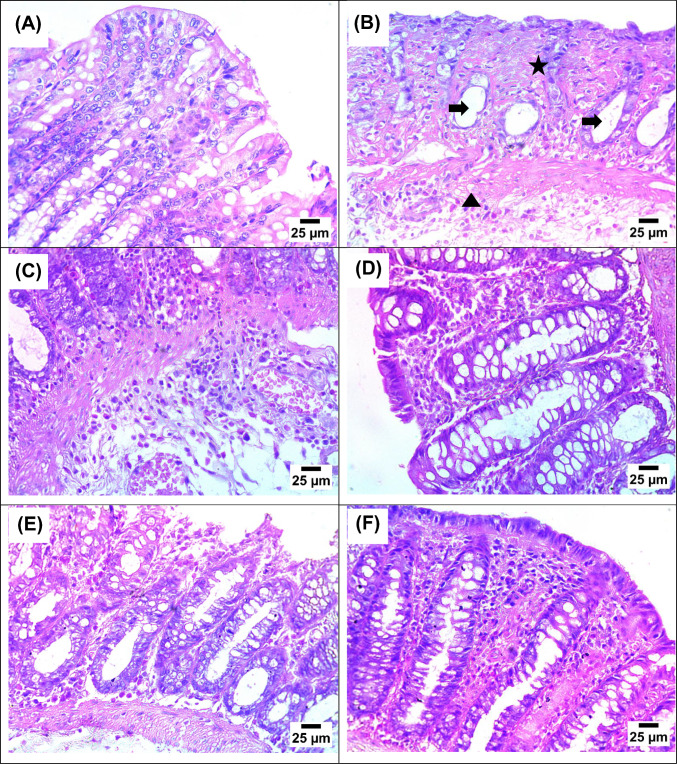
Histopathological appearance of colon tissue. **A**. photomicrograph of NC group showing normal histological structure of intestinal mucosa; **B**. photomicrograph of UC group showing necrosis in intestinal mucosa (star) with necrobiotic changes in intestinal gland (arrows), presence of submucosal infiltration by eosinophiles and lymphocytes (arrow head); **C**. photomicrograph of CT1-200 group showing infiltration of mucosa and submucosa by high number of inflammatory cells mainly eosinophiles and lymphocytes (arrow heads), presence of submucosal blood vessels congestion (arrow); **D**. photomicrograph of CT1-400 group showing infiltration of intestinal mucosa by mononuclear inflammatory cells (star); **E**. Photomicrograph of CT2-200 group showing eroded intestinal epithelium (arrow) with infiltration of intestinal mucosa by eosinophiles and lymphocytes (star); **F** photomicrograph of CT2-400 group showing infiltration of intestinal mucosa by mononuclear inflammatory cells (star) (Hematoxylin and Eosin stain)

The colon tissue of NC group showed the normal histological structure of intestinal mucosa (Fig. 5). Induction of UC revealed necrosis in intestinal mucosa with necrobiotic changes in the intestinal gland and, the presence of submucosal infiltration by eosinophiles and lymphocytes. Treatment of rats with CT1-200 showed infiltration of mucosa and submucosa by a high number of inflammatory cells mainly eosinophiles and lymphocytes, presence of submucosal blood vessels congestion. In the CT1 group receiving the 400 mg/kg dose, the intestinal lining showed infiltration by mononuclear inflammatory cells. For the CT2 treatments, the 200 mg/kg dose resulted in erosion of the intestinal epithelium along with the infiltration of both eosinophils and lymphocytes into the mucosa. In contrast, the higher 400 mg/kg dose of CT2 primarily caused infiltration by mononuclear inflammatory cells.

#### Effect on qPCR analysis of SIRT1, PPAR-γ, and GAPDH mRNA expression

**Fig. 6:**
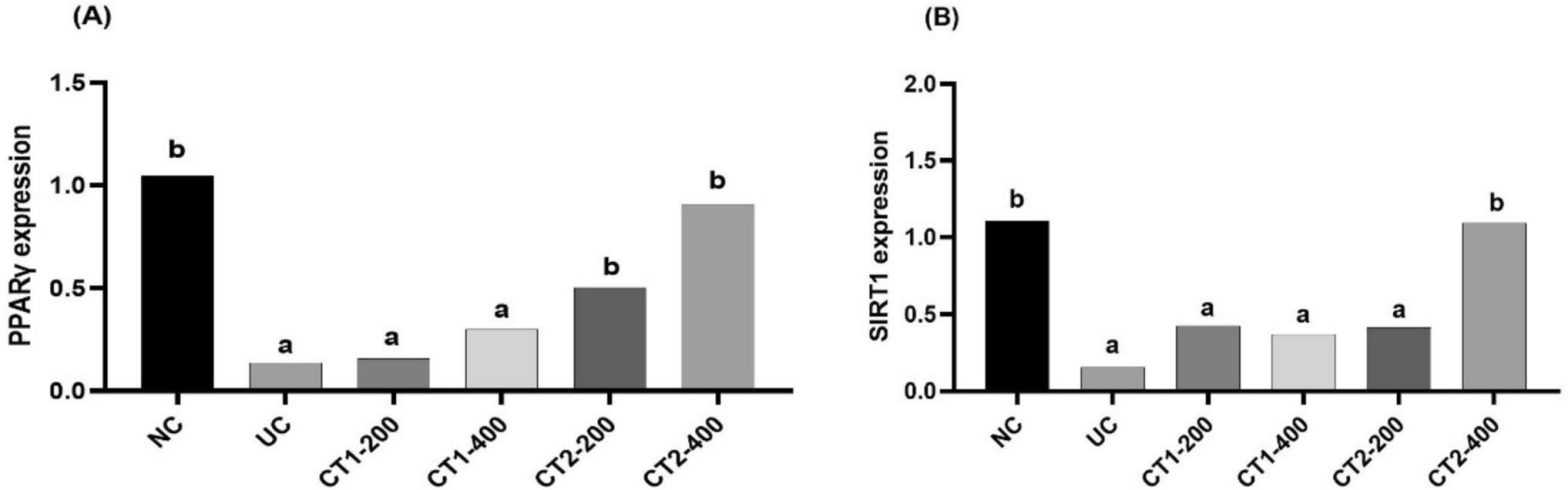
Effect on colon PPARγ and SIRT1 gene expression. Data presented as mean ± SEM (n = 6). ^a^Statistically significant from the NC group at *P* ≤ 0.05. ^b^Statistically significant from the UC group at *P* ≤ 0.05

UC group revealed significant decline in PPARγ and SIRT1mRNA expression in rat colon tissue (Fig. 6). However, on CT1 and CT2 treatment, significant increase was recorded in PPARγ and SIRT1mRNA expression at the two examined dose levels compared to UC group, with almost normal levels in CT-400 treated group.

### Phytochemical study

#### Untargeted HPLC–ESI–MS/MS analysis

**Fig. 7:**
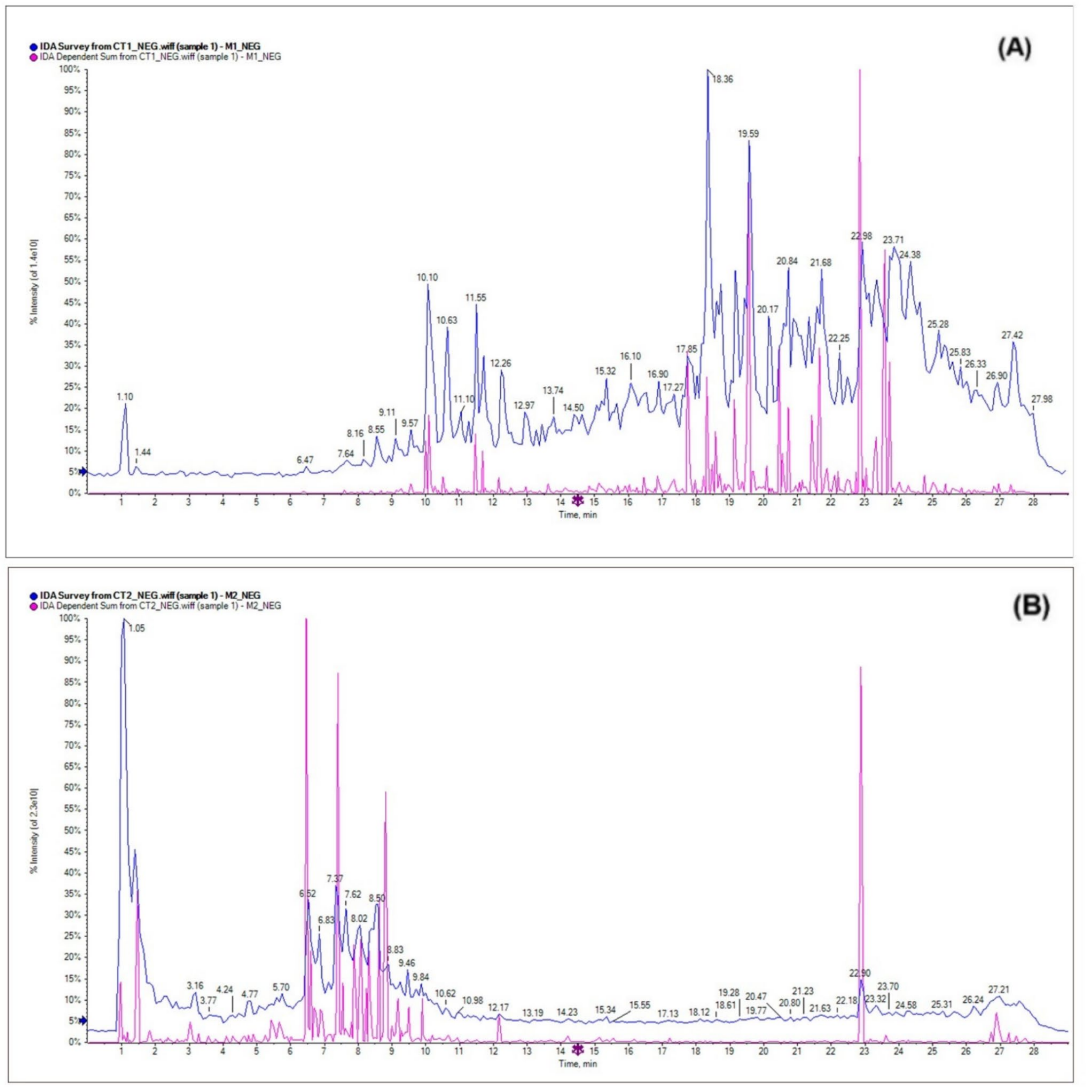
The total ion chromatograms (TIC) of CT1 (a) and CT2 (b)

CT1 and CT2 were studied using a negative mode of HPLC–ESI–MS/MS. Table 4 describes CT1 and CT2 constituents’ annotation and their complete data. The annotation was achieved by comparing the data from CT1 and CT2 with existing literature and the following databases: the European mass bank database (Horai et al. 2010), The Human Metabolome Database (HMDB) (Wishart et al. 2022), and The Global Natural Product Social Molecular Networking (GNPS) (Wang et al. 2016). Figure 7a and b depict the total ion chromatograms of CT1 and CT2, respectively.

**Table 4:** Annotated compounds of CT1 and CT2 using HPLC–ESI–MS/MS in negative mode

No	Rt. (min.)	CT1	CT2	Annotation	[M-H]^−^	Formula	MS/MS	References
1	1.13	−	+	Galatitol	181	C_6_H_14_O_6_	119, 101	(Wishart et al. 2022)
2	1.18	+	+	Caffeic acid ^ce^	179	C_9_H_8_O_4_	135, 117, 107	(Hassan et al. 2021; Wishart et al. 2022)
3	1.43	+	+	Quinic acid	191	C_7_H_12_O_6_	155, 135, 127, 119, 111	(Wishart et al. 2022)
4	1.47	+	+	Gallic acid ^be^	169	C_7_H_6_O_5_	125, 107	(El-Hawary et al. 2016b, a; Wishart et al. 2022)
5	1.71	+	+	Citric acid ^c^	191	C_6_H_8_O_7_	173, 111	(Hassan et al. 2021; Wishart et al. 2022)
6	2.84	+	+	Protocatechuic acid ^be^	153	C_7_H_6_O_4_	135, 117, 109	(El-Hawary et al. 2016b; Wishart et al. 2022)
7	4.03	+	+	Methyl gallate ^be^	183	C_8_H_8_O_5_	140, 124, 111	(El-Hawary et al. 2016b; Wishart et al. 2022)
8	4.73	+	+	Hydroxy benzoic acid ^b^	137	C_7_H_6_O_3_	121, 109	(El-Hawary et al. 2016b; Wishart et al. 2022)
9	5.47	−	+	Chlorogenic acid ^ce^	353	C_16_H_18_O_9_	191, 179, 161, 135	(Hassan et al. 2021; Wishart et al. 2022)
10	5.53	−	+	Isoquercitrin	463	C_21_H_20_O_12_	301, 271, 151	(Wishart et al. 2022)
11	5.77	+	+	Rutin ^ce^	609	C_27_H_30_O_16_	591, 299	(Wang et al. 2016; Hassan et al. 2021; Wishart et al. 2022)
12	5.85	+	+	Kaempferol coumaroyl hexoside	593	C_30_H_26_O_13_	285, 255	(Horai et al. 2010)
13	5.92	−	+	Quercetin pentoside ^b^	433	C_20_H_18_O_11_	300, 271, 255, 179	(Horai et al. 2010; El-Hawary et al. 2016a)
14	6.54	+	+	Quercetin deoxyhexoside ^c^	447	C_21_H_20_O_11_	357, 327, 301, 271, 151	(Horai et al. 2010; Wang et al. 2016)
15	7.43	−	+	Epigallocatechin coumarate	451	C_24_H_20_O_9_	289, 271, 209, 191, 179, 167, 125	(Wishart et al. 2022)
16	7.89	−	+	Eriodictyol hexoside	449	C_21_H_22_O_11_	287, 175, 151, 135	(Wang et al. 2016)
17	7.94	+	−	Vanillin ^e^	151	C_8_H_8_O_3_	136, 108	(Wishart et al. 2022)
18	8.04	+	+	Trihydroxy octadecenoic acid	329	C_18_H_34_O_5_	283, 229, 211, 183, 171, 139, 127	(Horai et al. 2010; Wang et al. 2016)
19	8.27	+	+	Vanillic acid ^b^	167	C_8_H_8_O_4_	152, 137, 124, 108	(El−Hawary et al. 2016b; Wishart et al. 2022)
20	8.34	+	+	Syringic acid ^ce^	197	C_9_H1_0_O_5_	153, 123, 119, 109	(Wishart et al. 2022)
21	8.37	+	+	Ferulic acid ^e^	193	C_10_H_10_O_4_	161, 157, 134	(Wishart et al. 2022)
22	8.41	−	+	Catechin ^e^	289	C_15_H_14_O_6_	255, 167, 123	(Wishart et al. 2022)
23	8.47	+	+	Coumaric acid ^ce^	163	C_9_H_8_O_3_	145, 119	(Hassan et al. 2021; Wishart et al. 2022)
24	8.61	+	+	Homogentisic acid	167	C_8_H_8_O_4_	123, 108	(Wishart et al. 2022)
25	9.04	+	+	Ellagic acid ^e^	301	C_14_H_6_O_8_	255, 179, 145, 121, 107	(Wishart et al. 2022)
26	9.136	+	+	Hydroxy dodecanedioic acid	245	C_12_H_22_O_5_	313 [M-H + HCOONa]^−^, 229, 201, 185, 157, 135	(Wishart et al. 2022)
27	9.46	+	+	Isosulochrin	331	C_17_H_16_O_7_	287, 269, 257, 219, 203, 175, 165, 151, 123, 108	(Wang et al. 2016)
28	9.5	−	+	Eriocitrin	595	C_27_H_32_O_15_	307, 287, 175, 150, 135	(Wishart et al. 2022)
29	9.83	+	+	Phloretin	273	C_15_H_14_O_5_	227, 189, 167, 151, 123, 119	(Horai et al. 2010; Wang et al. 2016)
30	10.12	+	+	Dihydroxy methoxy chalcone	269	C_16_H_14_O_4_	239, 200, 185, 157, 121	(Wishart et al. 2022)
31	10.16	+	+	3-Hydroxy-4,6-heptadiyne-1-yl 1-glucoside	285	C_13_H_18_O_7_	269, 255, 237, 179, 165, 123	(Wishart et al. 2022)
32	10.23	+	+	Isorhamnetin ^b^	315	C_16_H_12_O_7_	300, 271, 245, 231, 187, 158, 135	(Horai et al. 2010; El-Hawary et al. 2016b)
33	10.62	+	+	Methyluridine	257	C_10_H_14_N_2_O_6_	241, 227, 199, 174, 145, 135, 108	(Wishart et al. 2022)
34	10.89	+	+	Rosmarinic acid	359	C_18_H_16_O_8_	197, 179, 161, 123	(Horai et al. 2010; Wang et al. 2016)
35	10.96	+	+	Kaempferol hexoside ^c^	447	C_21_H_20_O_11_	347, 327, 285, 177	(Horai et al. 2010; Wang et al. 2016)
36	11.24	+	+	Kaempferol pentoside ^b^	417	C_20_H_18_O_10_	285, 255	(Horai et al. 2010; El-Hawary et al. 2016a)
37	11.53	+	−	Assamicain A or C ^c^	915	C_44_H_36_O_22_	457, 413, 358, 313, 257	(Wishart et al. 2022)
38	11.81	−	+	6```-hydroxylophirone B ([(2S,3R)-naringenin-(3β,3)-4,2`,4`-trihydroxychalcone] ^d^	525	C30H22O9	327, 271, 221, 161	(Kaewamatawong et al. 2002)
39	11.92	+	+	Luteolin hexoside ^c^	447	C_21_H_20_O_11_	429, 357, 327, 287, 257, 175	(Horai et al. 2010; Wang et al. 2016)
40	12.26	+	+	Rollipyrrole	287	C_16_H_20_N_2_O_3_	269, 257, 217, 203, 189, 175, 159, 136, 108	(Wishart et al. 2022)
41	12.26	−	+	Eriodictyol ^cd^	287	C_15_H_12_O_6_	241, 225, 217, 203, 151, 135, 107	(Wang et al. 2016; Wishart et al. 2022)
42	12.56	−	+	Quercetin 3,7-dimethyl ether ^d^	329	C_17_H_14_O_7_	285, 271, 255, 165, 123, 108	(Wang et al. 2016)
43	12.93	+	−	Hispidulin acetate	425	C_22_H_18_O_9_	325, 283, 268, 191, 176, 141	(Horai et al. 2010)
44	14.07	−	+	Padmatin ^d^	317	C_16_H_14_O_7_	298, 193, 173, 149, 119	(Minh et al. 2022)
45	15.2	+	+	Docosanol	325	C_22_H_46_O	253, 239, 197, 183, 170, 119	(Wang et al. 2016; Wishart et al. 2022)
46	15.23	−	+	3,5,7,4`,3``,5``,7``-Heptahydroxy-3`-O-4```-biflavanone ^d^	573	C_30_H_22_O_12_	555 [M-H-H_2_O]^−^, 303, 271, 225, 165	(Pegnyemb et al. 2005),
47	16.29	+	+	Hesperetin ^e^	301	C_16_H_14_O_6_	283, 215, 164, 151, 123	(Wishart et al. 2022)
48	16.53	+	−	Cyclolaudenol ^a^	439	C31H52O	395, 351, 333, 205, 191	(Eid 2013; Wishart et al. 2022)
49	16.86	+	+	Quercetin ^be^	301	C_15_H_10_O_7_	283, 215, 200, 172, 151, 123	(El-Hawary et al. 2016b; Wishart et al. 2022)
50	16.94	+	−	Biochanin A	283	C_16_H_12_O_5_	253, 240, 215, 200, 187, 161, 145, 121	(Horai et al. 2010)
51	17.23	+	−	Xanthohumol	353	C21H22O5	295, 269, 233, 177, 163	(Horai et al. 2010; Wang et al. 2016)
52	17.64	+	−	Lupeol ^a^	425	C_30_H_50_O	407, 205, 193	(Eid 2013; Wishart et al. 2022)
53	17.72	+	+	Luteolin ^ce^	285	C_15_H_10_O_6_	255, 223, 150, 133, 123, 108	(Wang et al. 2016)
54	18.23	+	−	Campesterol ^a^	399	C_28_H_48_O	381, 353, 257, 231, 165, 141, 123	(Eid 2013; Wishart et al. 2022)
55	18.45	+	+	Kaempferol ^be^	285	C_15_H_10_O_6_	255, 223, 165, 123, 119	(El-Hawary et al. 2016a; Wishart et al. 2022)
56	19.63	+	−	Linolenic acid	277	C_18_H_30_O_2_	259, 233, 165, 151, 141, 125, 107	(Horai et al. 2010)
57	20.15	+	−	Quercetin pentamethyl ether	371	C_20_H_20_O_7_	431 [M + CH3COOH-H], 311, 299, 283, 259, 229, 205	(Wishart et al. 2022)
58	20.22	+	+	1-Methoxy-3-(4-hydroxyphenyl)-2E-propenal 4’-glucoside	325	C_16_H_22_O_7_	310, 266, 241, 175, 148, 123	(Wishart et al. 2022)
59	20.59	+	+	Naringenin ^ce^	271	C_15_H_12_O_5_	253, 163, 151, 144	(Hassan et al. 2021; Wishart et al. 2022)
60	21.33	+	−	(α/ β) amyrin ^b^	425	C_30_H_50_O	407, 300, 231, 167, 123	(El-Hawary et al. 2016b; Wishart et al. 2022)
61	21.66	+	−	18-Hydroxyeicosatetraenoic acid	319	C_20_H_32_O_3_	399 [M + Br]^−^, 257, 229, 165	(Wishart et al. 2022)
62	22.92	+	+	Neotussilagolactone	343	C_21_H_28_O_8_	299, 217, 203, 163	(Wishart et al. 2022)
63	22.95	+	−	Palmitic acid ^c^	255	C_16_H_32_O_2_	237	(Hassan et al. 2021; Wishart et al. 2022)
64	22.96	+	+	13-Acetylphorbol	405	C_22_H_30_O_7_	345, 191, 167	(Wishart et al. 2022)
65	23.04	+	−	β-sitosterol ^a^	413	C_29_H_50_O	395, 385, 369, 257, 176	(Eid 2013; Wishart et al. 2022)
66	24.85	+	−	Baicalein	269	C_15_H_10_O_5_	251, 241, 223, 213, 207, 197, 169	(Wang et al. 2016)

^a^: Formerly recorded in *C. tetragona*

^b^: Formerly recorded in *Crassula* genus

^c^: Formerly recorded in the *Crassulaceae* family

^d^: Isolated in this study

^e^: Quantified in MRM-LC–ESI–MS/MS section

A total of 66 constituents were annotated across both extracts: 53 in CT1, 51 in CT2, with 38 compounds common to both. The annotated compounds included 30 flavonoids (13 flavones, 4 flavonols, 2 flavanes, 3 chalcones, 7 flavanones, and a biflavanone), 13 phenolic acid derivatives, 6 fatty acids, 2 tannins, 2 carboxylic acids, 2 steroids, 2 triterpenes, a sugar alcohol, a phenol, a pyrimidine nucleoside, a pyrroline derivative, an alcohol, a glycoside, a lipid, a phytosterol and a terpene.

##### Previously reported compounds

Four compounds (two steroids: cyclolaudenol **48** and campesterol **54**; a phytosterol: β-sitosterol **65** and a triterpene: lupeol **52**) were formerly recorded in *C. tetragona* and were detected here exclusively in CT1 (Eid 2013; Wishart et al. 2022). Eleven compounds (mainly phenolic acids: gallic acid **4**, protocatechuic acid **6**, methyl gallate **7**, hydroxybenzoic acid **8**, vanillic acid **19**, isorhamnetin **32**, kaempferol pentoside **36**, quercetin **49**, kaempferol **55**; and a triterpene: (α/β)-amyrin **60** were previously reported in other *Crassula* species (*C. arborescens, C. capitella*) In this study, (α/ β) amyrin **60** was detected only in CT1 and quercetin pentoside **13** was detected only in CT2 (Horai et al. 2010; El-Hawary et al. 2016b, a; Wishart et al. 2022). Fourteen of the annotated compounds were previously reported in the *Crassulaceae* family (Hassan et al. 2021). The following *Crassulaceae* phenolic compounds: caffeic acid **2**, citric acid **5**, rutin **11**, quercetin deoxyhexoside **14**, syringic acid **20**, coumaric acid **23**, kaempferol hexoside **35**, luteolin hexoside **39**, luteolin **53**, and naringenin **59** were detected in both extracts. While assamicain A or C **38** and palmitic acid **63** were detected only in CT1 and chlorogenic acid **9** and eriodictyol **41** were detected only in CT2 (Horai et al. 2010; Wang et al. 2016; Wishart et al. 2022).

##### Novel compounds

Thirty-seven compounds were annotated here for the first time from *Crassula*. Beside the abovementioned phenolic compounds, the following phenolics were detected in both extracts: kaempferol coumaroyl hexoside **12**, ferulic acid **21**, homogentisic acid **24**, ellagic acid **25**, isosulochrin **27**, phloretin **29**, dihydroxy methoxy chalcone **30**, Rosmarinic acid **34**, and hesperetin **47**. Moreover vanillin **17**, hispidulin acetate **43**, biochanin A **50**, xanthohumol **51**, quercetin pentamethyl ether **57**, and baicalein **66** were detected only in CT1. While isoquercitrin **10**, epigallocatechin coumarate **15**, eriodictyol hexoside **16**, catechin **22**, eriocitrin **28**, 6```-hydroxylophirone B **38**, quercetin 3,7-dimethyl ether **42**, padmatin **44**, and 3,5,7,4`,3``,5``,7``-Heptahydroxy-3`-O-4```-biflavanone **46** were detected only in CT2 (Horai et al. 2010; Wang et al. 2016; Wishart et al. 2022). The three fatty acids: trihydroxy octadecenoic acid **18**, hydroxy dodecanedioic acid **26**, and 3-hydroxy-4,6-heptadiyne-1-yl 1-glucoside **31** were detected in both extracts (Horai et al. 2010; Wang et al. 2016; Wishart et al. 2022). Quinic acid **3**, methyluridine **33**, rollipyrrole **40**, docosanol **45**, 1-methoxy-3-(4-hydroxyphenyl)-2E-propenal 4’-glucoside **58**, neotussilagolactone **62**, and 13-acetylphorbol **64** were detected in both extracts. The sugar alcohol, galatitol **1** was detected only in CT2 (Horai et al. 2010; Wang et al. 2016; Wishart et al. 2022).

Caffeic acid **2**, gallic acid **4**, protocatechuic acid **6**, methyl gallate **7**, chlorogenic acid **9**, rutin **11**, vanillin **17**, syringic acid **20**, ferulic acid **21**, catechin **22**, coumaric acid **23**, ellagic acid **25**, hesperetin **47**, quercetin **49**, luteolin **53**, kaempferol **55**, naringenin **59** were further quantified in the extracts via MRM-LC–ESI–MS/MS.

The isolation of 6```-hydroxylophirone B **38**, eriodictyol **41**, quercetin 3,7-dimethyl ether **42**, padmatin **44**, 3,5,7,4`,3``,5``,7``-Heptahydroxy-3`-O-4```-biflavanone **46** from CT2 was described under isolation section.

#### Quantitative determination of phenolics using MRM-LC–ESI–MS/MS

**Fig. 8:**
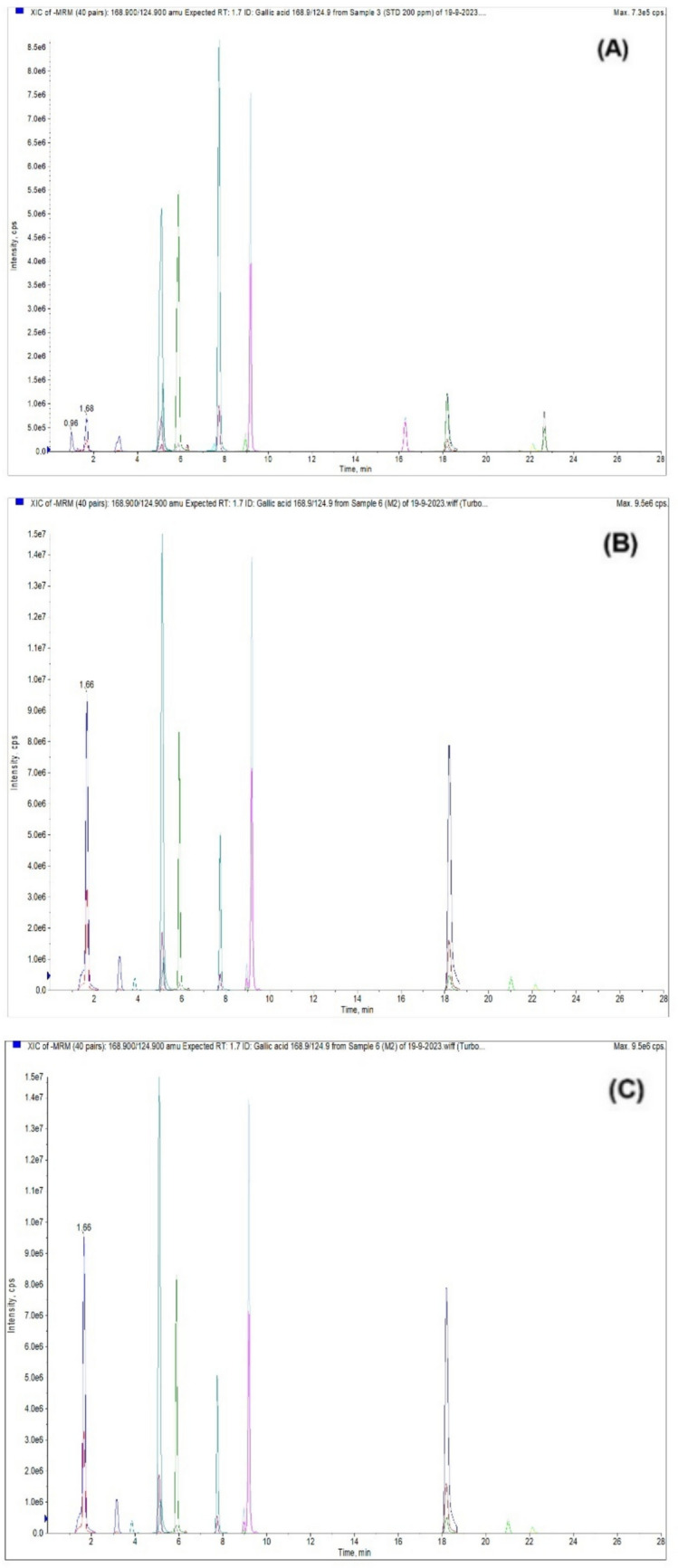
Extracted Ion Chromatograms (XICs) of the standard mixture (200 ppb) (a), CT1 (b), and CT2 (c)

Phenolic compounds being major constituents in CT1 and CT2, their quantification was undertaken in both extracts. Figure 8 displays Extracted Ion Chromatograms (XICs) of the standard mixture (200 ppb), CT1, and CT2. The quantified levels of the phenolic compounds found in both extracts are summarized in Table 5.

**Table 5:** The concentrations of the phenolic constituents in CT1 and CT2

Retention Time (Min.)	Phenolic, Q1/Q3 (m/z)	Conc. (μg/g)	
		CT1	CT2
1.67	Gallic acid 168.9/124.9	76.38	334.63
5.86	Caffeic acid 178/135	9.43	30.72
9.18	Rutin 609/299.9	0.20	51.64
7.73	Coumaric acid 162.9/119	13.16	13.20
7.51	Vanillin 151/136	52.03	ND
21.02	Naringenin 271/151	431.03	1788.02
18.2	Quercetin 301/151	66.82	190.24
9.03	Ellagic acid 301/145	2.76	14.38
3.15	Protocatechuic acid (3.4-Dihydroxybenzoic acid) 152.9/109	15.35	76.12
22.65	Hesperetin 301/164	0.08	0.05
18.2	Cinnamic acid 146.9/102.6	ND	ND
5.08	Methyl gallate 183/124	41.72	56.73
22.12	Kaempferol 284.7/93	5.34	35.37
8.95	Ferulic acid 192.8/133.9	3.64	8.32
6.29	Syringic acid 196.9/122.8	1.95	11.78
21.52	Apigenin 269/151	ND	ND
5.11	Catechin 288.8/244.9	ND	11.13
18.19	Luteolin 284.7/132.9	3.15	13.08
16.27	Daidzein 253/132	ND	ND
5.16	Chlorogenic acid 353/191	ND	15.82

ND, not detected

Of the twenty phenolic standards, 15 were detected and quantified in CT1, while 16 were detected and quantified in CT2. Naringenin (431.03 and 1788.02 μg/g), gallic acid (76.38 and 334.63 μg/g), and quercetin (66.82 and 190.24 μg/g) were the most abundant phenolics in CT1 and CT2, respectively. Vanillin was exclusively detected in CT1, while catechin and chlorogenic acid were found only in CT2. The concentrations of all detected phenolics were significantly higher in CT2 compared to CT1, except for hesperetin and coumaric acid, which exhibited comparable levels.

#### Isolated compounds

**Fig. 9:**
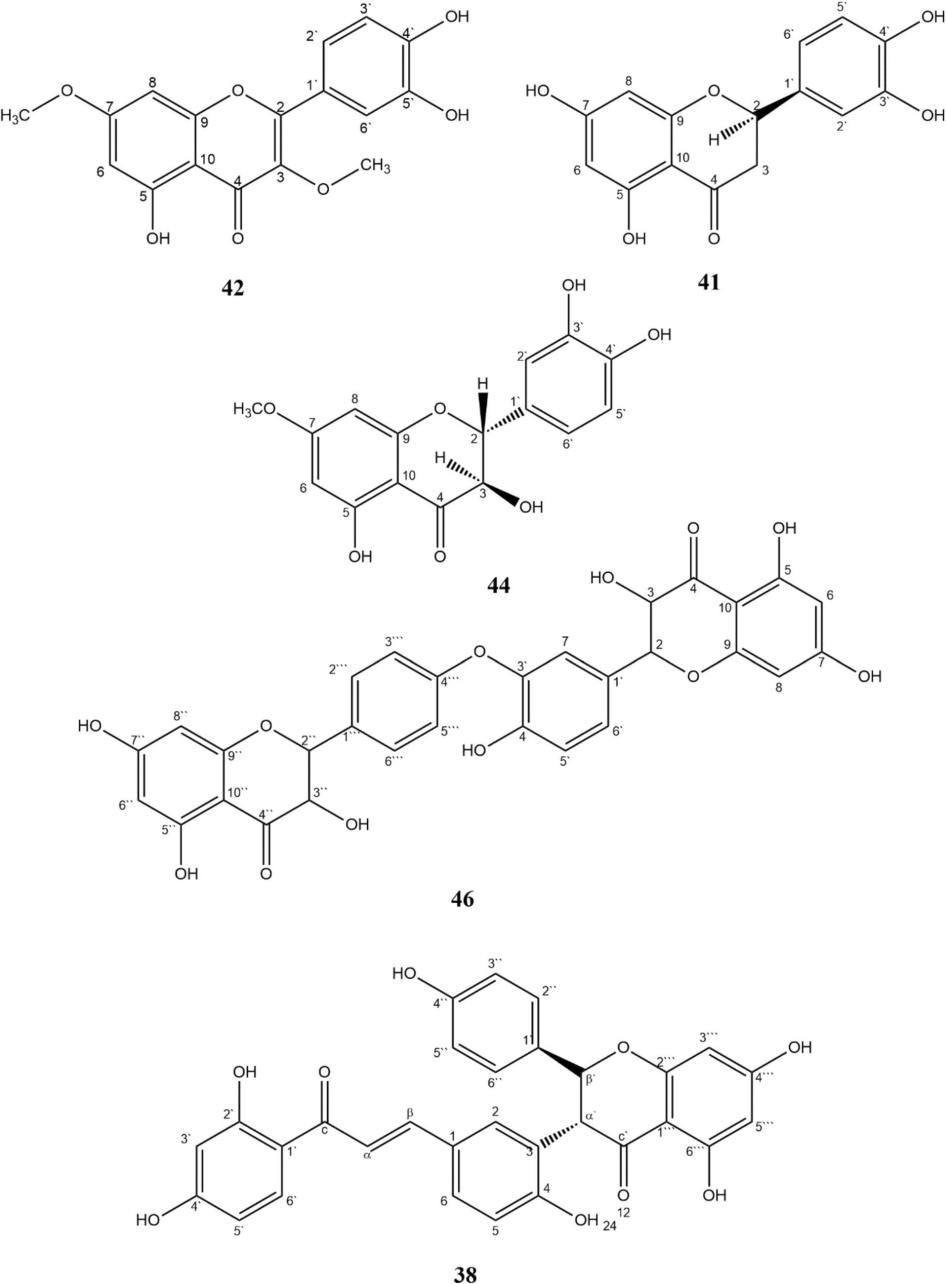
Isolated compounds of CT2

Combined column chromatography and preparative paper chromatography of the bioactive extract CT2 yielded five phenolic compounds (Fig. 9). According to their spectroscopic data (ESI–MS, ^1^H and ^13^C-NMR) and comparison with reported data, they were characterized as quercetin 3,7-dimethyl ether (3',4',5-trihydroxy-3,7-dimethoxyflavone) **42** (Nantapap et al. 2017), (2S)-eriodictyol (3',4',5,7-tetrahydroxyflavanone) **41** (Buranasudja et al. 2022), padmatin **44** (Minh et al. 2022), 3,5,7,4`,3``,5``,7``-heptahydroxy-3`-O-4```-biflavanone **46** (Sievers et al. 1992; Pegnyemb et al. 2005), 6```-hydroxylophirone B ([(2S,3R)-naringenin-(3β,3)-4,2`,4`-trihydroxychalcone] **38** (Kaewamatawong et al. 2002). As far as we know, this is the first report of these compounds being isolated from *C. tetragona* L.

## Discussion

The main goal of this research was to analyse the phytochemical profile of the poorly studied plant *Crassula tetragona* L. and to evaluate its potential effectiveness in treating ulcers. Our outcomes reveal a diverse phytochemical composition, with a total of 66 constituents identified across the n-hexane (CT1) and 70% aqueous methanolic (CT2) extracts using HPLC–ESI–MS/MS analysis in negative ion mode. The novelty of this research is underscored by the discovery of 37 compounds that had never before been documented in the *Crassula* genus.

The quantitative analysis revealed significant differences in phenolic compound profiles between CT1 and CT2. CT2 exhibited a higher abundance of most phenolics, suggesting that the aqueous methanolic extraction method effectively extracted a broader range of polar compounds, including flavonoids and phenolic acids. The identification of compounds like naringenin, gallic acid, and quercetin, known for their anti-inflammatory and antioxidant properties, further supports the potential therapeutic effect of *C. tetragona*.

The exclusive presence of 13 compounds, including galatitol **1**, chlorogenic acid **9**, isoquercitrin **10**, quercetin pentoside **13**, epigallocatechin coumarate **15**, eriodictyol hexoside 16, catechin **22**, eriocitrin **28**, 6```-hydroxylophirone B **38**, eriodictyol **41**, quercetin 3,7-dimethyl ether **42**, padmatin **44**, 3,5,7,4`,3``,5``,7``-heptahydroxy-3`-O-4```-biflavanone **46** in CT2 suggests a potential correlation between these compounds and the observed biological activity. These compounds are well-known to possess anti-inflammatory and antioxidant properties, which may contribute to the observed anti-ulcerative effects of CT2 (Ambriz-Pérez et al. 2016; Zhang and Tsao 2016; Kumar and Goel 2019; Baranwal et al. 2022; Azeem et al. 2023). The isolation and characterization of five phenolic compounds (6```-hydroxylophirone B, eriodictyol, quercetin 3,7-dimethyl ether, padmatin, and 3,5,7,4',3'',5'',7''-Heptahydroxy-3'-O-4''-biflavanone) from CT2 provides a foundation for further investigation into their specific anti-ulcerative mechanisms and potential as lead compounds for drug development.

Ulcerative colitis is a chronic, relapsing gut disease driven by the complex interaction of genetic, environmental, and immune factors, leading to inflammation, oxidative stress, and eventual tissue damage, including ulceration, bleeding, and necrosis (Cho and Brant 2011; Rana et al. 2014). The current study demonstrated for the first time the preventive effects of *C. tetragona* L CT1 and CT2 against acetic acid (AA)-induced colitis in rats. Acetic acid-induced colitis is a well-researched technique for developing an animal model of UC because it mimics the human disease in that involves localized involvement, destruction to the intestinal epithelium, and an increase in inflammatory mediators (Elson et al. 1995).

Consistent with established models, the rectal AA administration successfully created a severe colitis state, resulting in a considerable increase in rat colon weight, severe edema, and pronounced macroscopic lesions and ulceration. Histologically, the colonic mucosa of the AA group showed extensive disruption, including erosion, ulceration, congestion, necrosis, and massive inflammatory cell infiltration (Randhawa et al. 2014; Abdel-Rahman et al. 2015; Ali et al. 2017). Pre-treatment with CT1 and CT2 reduced colitis in a dose-dependent manner in comparison to colitis animals, with the CT2 treated groups exhibiting the most noticeable results. It was shown that both treatment groups significantly reduced the gross lesion score and reversed the colon tissue’s histological deterioration.

Our biochemical analysis provides clear evidence of *C. tetragona*'s anti-inflammatory action. In the colitis control group, there was a predictable and marked elevation of C-reactive protein (CRP) and Calprotectin (CALP). Both are established biomarkers for UC activity, reflecting the severity of intestinal mucosal inflammation and leukocyte activation (DʼHaens et al. 2012; Lobatón et al. 2013; Brookes et al. 2018). Treatment with CT1 and CT2 resulted in a substantial decrease in these elevated CALP and CRP levels, further validating the extracts’ ability to suppress the inflammatory process in the colon. The colon inflammatory state in the AA group was also characterized by a significant imbalance of cytokines: high levels of the pro-inflammatory TNF-α and IL-6 and low levels of the anti-inflammatory IL-10 (Fan et al. 2009; Saber et al. 2019). The elevated TNF-α and IL-6 are central to UC pathophysiology, driving inflammation, NF-κB activation, and ROS generation (Liu 2011; Mitsialis et al. 2020). Conversely, IL-10 is critical for maintaining remission (Wang et al. 2020). Both CT1 and CT2 demonstrated potent anti-inflammatory effects by significantly elevating IL-10 and inhibiting the production of both TNF-α and IL-6.

Furthermore, our data confirmed a state of severe oxidative stress in the colitis control group. The inflammatory surge and subsequent release of Reactive Oxygen Species ROS by activated leukocytes deplete the colon’s defence mechanisms (Balmus et al. 2016). This was evidenced by a significant rise in Malondialdehyde (MDA), a marker of lipid peroxidation, and a concomitant depletion of the antioxidant capacity, specifically a reduction in GSH levels and SOD activity (Ferrat et al. 2019). Critically, CT1 and CT2 (especially CT2) dramatically restored this antioxidant defence, effectively reducing MDA while simultaneously replenishing both GSH and SOD activity. These findings are consistent with the known antioxidant qualities of the Crassulaceae family (Eid et al. 2018).and confirm that *C. tetragona*'s anti-colitis efficacy is strongly linked to its antioxidant properties.

This study provides the first evidence that the anti-ulcerative effect of CT1 and CT2 involves the SIRT1 and PPARγ signaling pathways. SIRT1, a key regulator of inflammation, cell death, and antioxidant defence (Carafa et al. 2016), was markedly downregulated in the AA induced colitis model, consistent with previous clinical and preclinical research in IBD (Caruso et al. 2014). The ability of SIRT1 to deacetylate and suppress the pro-inflammatory transcription factor NF-κB makes it a vital therapeutic target (Han et al. 2019). Treatment with CT1 and CT2 successfully reversed this downregulation, significantly boosting SIRT1 expression, suggesting that the anti-UC action of these extracts is mediated, at least in part, by activating this pathway. Similarly, the nuclear receptor PPARγ is essential for regulating inflammation and maintaining the mucosal barrier in the colon (Desreumaux et al. 2001; Fang et al. 2021). Its expression is often low in UC patients, and agonists are known to reduce colitis symptoms. Our results show that CT1 and CT2 significantly elevated PPARγ expression in the colitis model. By promoting PPARγ, the extracts likely suppress downstream inflammatory factors and enhanced the integrity of the colon tissue, findings that align with the known protective role of PPARγ against AA-induced colitis (Zeng et al. 2011; Sethuraman et al. 2015).

Despite the strong evidence for the anti-ulcerative potential of *C. tetragona* L., this study has several limitations that must be addressed. While we have established the involvement of the SIRT1 and PPARγ pathways, the precise molecular mechanism by which the crude CT1 and CT2 extracts activate these receptors remains unclear. Future studies require isolating pure compounds to directly test their affinity and signaling actions. The observed effect is based on the crude extracts CT1 and CT2. We were unable to test the anti-ulcerative activity of the individual isolated compounds in vivo. Therefore, the specific compound(s) responsible for the superior efficacy of CT2 are yet to be definitively confirmed. This study did not investigate the oral bioavailability or metabolic fate of the identified phenolic compounds in the rat model. Understanding how these compounds are absorbed, distributed, and metabolized is crucial for predicting their clinical efficacy. While natural products often have low toxicity, a comprehensive toxicity and long-term safety assessment of the CT2 extract is required before clinical application can be considered. The AA-induced colitis model is an acute inflammation model. To fully understand the plant’s relevance for human UC, which is a chronic, relapsing disease, testing the extracts in a chronic colitis model would be essential.

## Conclusion

This investigation provides valuable and novel insights into the phytochemical profile of *C. tetragona* L., revealing a diverse array of compounds, including 37 novel constituents. The pronounced difference between the CT1 and CT2 extracts underscores the critical role of the extraction method in recovering bioactive compounds, with CT2 demonstrating superior yield and biological activity. For the first time, this study reveals the potent anti-ulcerative benefits of *C. tetragona* L., primarily the CT2 extract, against AA-induced colitis. Its protective effect is achieved through a multi-target mechanism: reducing intestinal inflammation (decreasing TNF-α and IL-6, increasing IL-10, and lowering CRP/CALP, combating oxidative stress (restoring GSH and SOD while reducing MDA), and modulating key therapeutic pathways SIRT1 and PPARγ. Our data strongly support the use of *C. tetragona* L. (specifically CT2) as a promising candidate for developing novel therapies for ulcerative colitis.

## Conflict of Interest

The authors certify that they have no competing financial or personal interests to disclose.

## Ethics approval

The Ethical Committee for Medical Research at the National Research Centre in Egypt formally approved the entire research protocol (Approval No.: MREC-13060133).

## Data Availability

No datasets were generated or analysed during the current study.
